# Utilization of maternal health services and its determinants: a cross-sectional study among women in rural Uttar Pradesh, India

**DOI:** 10.1186/s41043-019-0173-5

**Published:** 2019-05-27

**Authors:** Ranjana Singh, Sutapa B. Neogi, Avishek Hazra, Laili Irani, Jenny Ruducha, Danish Ahmad, Sampath Kumar, Neelakshi Mann, Dileep Mavalankar

**Affiliations:** 1 Indian Institute of Public Health, Delhi, India; 2 Population Council, New Delhi, India; 3 Braintree Global Health, Cambridge, MA USA; 4 Centre for Research and Action, Faculty of Health, University of Canberra, Bruce, Canberra, Australia; 5 Public Health Foundation of India, Plot 47, Sector 44, Institutional Area, Gurgaon, Haryana 122002 India; 6 Indian Institute of Public Health, Gandhinagar, India; 7 Community and Rural Development and Agriculture Departments, Shillong, Meghalaya India; 8 Rajiv Gandhi Mahila Vikas Pariyojna, Lucknow, India

**Keywords:** Utilization, Antenatal care, Delivery, Postnatal care, Marginalization, Self-help group (SHG)

## Abstract

**Background:**

Proper utilization of antenatal and postnatal care services plays an important role in reducing the maternal mortality ratio and infant mortality rate. This paper assesses the utilization of health care services during pregnancy, delivery and post-delivery among rural women in Uttar Pradesh (UP) and examines its determinants.

**Methods:**

Data from a baseline survey of UP Community Mobilization (UPCM) project (2013) was utilized. A cross-sectional sample of currently married women (15 to 49 years) who delivered a baby 15 months prior to the survey was included. Information was collected from 2208 women spread over five districts of UP. Information on socio-demography characteristics, utilization of antenatal care (ANC), delivery and postnatal care (PNC) services was collected. To examine the determinants of utilization of maternal health services, the variables included were three ANC visits, institutional delivery and PNC within 42 days of delivery. Separate multilevel random intercept logistic regressions were used to account for clustering at a block and gram panchayat level after adjusting for covariates.

**Results:**

Eighty-three percent of women had any ANC. Of them, 61% reported three or more ANC visits. Although 68% of women delivered in a health facility, 29% stayed for at least 48 h. Any PNC within 42 days after delivery was reported by 26% of women. In the adjusted analysis, women with increasing number of contacts with the health worker during the antenatal period, women exposed to mass-media and non-marginalized women were more likely to have at least three ANC visits during pregnancy. Non-marginalized women and women with at least three ANC visits were more likely than their counterparts to deliver in an institution. Contacts with health worker during pregnancy, marginalization, at least three ANC visits and institutional delivery were the strong determinants for utilization of PNC services. Self-help group (SHG) membership had no association with the utilization of maternal health services.

**Conclusions:**

Utilization of maternal health services was low. Contact with the health worker and marginalization emerged as important factors for utilization of services. Although not associated with the utilization, SHGs can be used for delivering health care messages within and beyond the group.

## Background

Globally, there has been a decline in the maternal mortality ratio (MMR) by 44% over the last 25 years, from 385 maternal deaths per 100,000 live births in 1990 to an estimated 216 maternal deaths per 100,000 live births in 2015 [1]. Approximately 99% of the global maternal deaths in 2015 took place in developing regions, with sub-Saharan Africa alone accounting for roughly 66% followed by Southern Asia. Nigeria and India are the two countries which are estimated to account for over one third of all maternal deaths worldwide in 2015, with an approximate 58,000 maternal deaths (19%) and 45,000 maternal deaths (15%), respectively [1].

The MMR of India is declining; it has come down from 212 in 2007–2009 to 167 in 2011–2013 [2]. However, there is a long way to achieve the target mentioned in the SDG goals. In India, there are wide variations in MMR across different states, ranging from 300 in Assam to 61 in Kerala [2]. Among the northern states, Uttar Pradesh has the highest MMR of 285 maternal deaths per 100,000 live births. Almost 70% of the maternal deaths happen within the age group of 20 to 29 years which is the age group where fertility rates are also very high [2]. It was reported that about 38% of maternal deaths were caused by haemorrhage, 11% due to sepsis and 5% due to obstructed labor [3], majority of which can be prevented if women regularly go for antenatal care (ANC), deliver in an institution and utilize postnatal care services.

It is well known and widely accepted that use of maternal health services reduces maternal morbidity and mortality [4–7]. Utilization of maternal health services is influenced by multiple factors and this required focused attention. A study done in Madhya Pradesh found an association between use of ANC and factors like women’s education, household’s standard of living, caste and religion [4]. Various studies in India have concluded socio-economic factors and service delivery environment as important determinants influencing maternal health services [5, 6]. In another study in India, proportion of institutional deliveries among women from low socio-economic status was just 13% as compared to 84% among highest wealth quintile [7]. It has also been shown that ANC is the gateway for other healthy behaviors adopted during and after pregnancy, like institutional delivery, providing newborn care, exclusive breastfeeding, complimentary feeding and many more [8].

Use of ANC increases the likelihood of skilled attendance at delivery, which later increases the use of postnatal care (PNC). Women having received at least one ANC during pregnancy had 3.52 times higher odds of receiving skilled attendance at delivery than women who did not receive any ANC [9]. Skilled attendance during the birth of all babies is considered to be crucial for reducing maternal and infant mortality and morbidity, especially in poor-resource countries [10]. With increase in institutional delivery, the maternal mortality and newborn mortality is expected to decline due to the presence of skilled birth attendants, supported by the essential infrastructure and referral services when required. Various studies and surveys in India have documented a rise in proportion of institutional deliveries since the launch of the conditional cash transfer scheme (Janani Suraksha Yojana, JSY); however, the studies could not find an association between institutional birth proportion and MMR [11–13].

A Cochrane review showed that community-based interventions are effective in significantly reducing maternal and neonatal morbidity and mortality [14]. In India, there are existing women groups in the community, called self-help groups (SHG), which can be used to create health-related awareness within and outside the group in the community. A SHG usually consists of 10–20 women living in the same village, who come together and agree to save a specific amount periodically. The savings of all SHG members are combined and deposited in a bank or a co-operative organization from where members may borrow money from the pooled account in case of requirement as loans. The main aim of these SHGs is income generation which helps to raise poor families out of poverty. Members of the SHGs meet regularly for various issues related to transactions, training and local problems. The platform of SHG can also be used to spread health-related promotion and preventive messages within and outside the group in the community. Studies have shown a positive impact of presence of SHGs on various health-related outcomes. In a study in India, it was observed that women from villages with SHGs are more likely to deliver in an institution and are more aware about family planning practices and their utilization is better [15]. A cluster randomized trial conducted in an indigenous community of Odisha and Jharkhand states of India found a positive association between presence of SHG and likelihood of newborn survival within first 6 weeks [16].

Uttar Pradesh (UP) is India’s fourth largest state and the country’s most populated state contributing 16% to the national population. The state comprises 75 districts and 820 development blocks. The population of the state is primarily rural (78%) [17]. Rajiv Gandhi Mahila Vikas Pariyojana (RGMVP) has developed a federated SHG structure in UP. The model comprises an institution that imbibes collective ownership, efficiency, equality, transparency and a strong sense of voluntary spirit.

Against this backdrop, the paper aimed to assess the utilization of maternal health services in rural UP. The objectives were to determine the proportion of women who completed three antenatal check-ups, had institutional deliveries and had postnatal check-up. We also analysed the determinants of utilization of maternal health services in the rural community with focus on SHG membership and marginalization.

## Methods

Data for the present paper was derived from a baseline survey of an intervention study in which RGMVP was working in ten blocks of eight districts of UP. RGMVP is a rights-based organization that works for poverty reduction, women’s empowerment and rural development in UP. Among these districts, intervention was supposed to be implemented in one block each from the six districts, and in two blocks each from the remaining two districts. RGMVP covered 100 gram panchayats (GPs) in these ten blocks of eight districts. This was a cross-sectional survey conducted in 2013 in the rural areas of five selected districts of UP: Raebareli, Hardoi, Mirzapur, Maharajganj and Sultanpur. The selection of districts was based on geographical diversity and duration of the existence of SHGs. A total of 15 blocks were selected with at least two from each of the districts [18]. This study protocol was reviewed and approved by the Institutional Review Board of the Population Council, New York.

To identify the eligible women from SHG households, house listing of the SHG members was done in all the villages of listed GPs of the 15 selected blocks. To identify eligible women from non-SHG households in the intervention area, a listing of non-SHG households was done in the neighbourhood of SHG households in each selected village/purva to ensure similar socio-economic characteristics of women in both the groups. Currently married women in the age group of 15 to 49 years and those who delivered a baby during 15 months prior to the survey were eligible to be included in the study. A period of 15 months was selected in order to minimize the recall bias. Information on socio-demographic characteristics, ANC, delivery and PNC was collected from 1729 women from SHG households and 479 women from non-SHG households during the survey. Informed consent for participation in the study was obtained from each participant in the local language by trained interviewers before start of the interviews.

Descriptive statistics were used to present socio-economic, demographic and household characteristics for all households, and also for SHG and non-SHG households separately. Socio-economic status of households, wealth index, was computed through principal component analysis using the variables of household amenities, assets and durables [18].

To find out the determinants of utilization of maternal health services, three outcome variables were considered: at least three ANC visits during pregnancy, institutional delivery (delivery in a public/private health facility) and any PNC within 42 days of delivery. Separate multivariable models were run for each of the three outcome indicators. The covariates adjusted in the model included age of women, type of family, women working to earn, mass-media exposure, number of contacts with the health worker during pregnancy, SHG membership and marginalization. SHG membership was present either if a target woman or one of her household member was a SHG member. The level of marginalization was defined using three indicators viz. ability to read or write, caste and socio-economic status (SES) of the household. Women belonging to scheduled caste or scheduled tribe (SC/ST) category, unable to read or write and belonging to the last quintile of SES were considered in the category of most marginalized. Non-marginalized women were those who had none of the mentioned factors. Women having any one and any two of the mentioned factors are categorized as having any one form of marginalization and any two form of marginalization, respectively. To take into account the clustering of women, where women were nested within GPs and GPs were nested within blocks, multilevel random intercept logistic regression was used for each of the three outcomes. Results of this were presented in the form of odds ratios (ORs) and 95% confidence intervals (CI).

## Results

### Household characteristics

Household socio-demographic characteristics are described in Table 1. Majority of them were Hindus (93%) and belonged to scheduled caste/tribe (53%). Majority of the households had a water pump as the source of drinking water (91%) and practiced open defecation (92%). Almost half of the participants (48%) had not attended school at all. These profiles were also looked at separately for women from SHG households and non-SHG households. It was observed that significantly higher proportion of SHG households were from SC/ST category and from lower wealth quintiles as compared to non-SHG households. SHG households had a bigger family size (mean = 7.4, SD = 3.1) when compared with non-SHG households (mean = 6.8, SD = 3.0).

**Table 1:** Profile of the surveyed households and the eligible woman

Variables	All women *n* = 2208	Women from SHG HH *n* = 1729	Women from Non-SHG HH *n* = 479	*p* value
Household characteristics				
Religion (%)				0.865
Hindu	2046 (92.7)	1603 (92.7)	443 (92.5)	
Caste (%)				< 0.001
SC/ST	1172 (53.0)	951 (54.9)	221 (46.1)	
Other backward caste	772 (35.0)	576 (33.3)	196 (40.9)	
Others	264 (12.0)	202 (11.7)	62 (13.0)	
Type of family (%)				0.003
Nuclear	992 (44.9)	744 (43.0)	248 (51.8)	
Non-nuclear	1216 (55.1)	985 (57.0)	231 (48.2)	
Drinking water (%)				0.433
Piped	54 (2.4)	40 (2.3)	14 (2.9)	
Pump	2006 (90.9)	1578 (91.3)	428 (89.4)	
Other sources	148 (6.7)	111 (6.4)	37 (7.7)	
Toilet facility (%)				0.041
Flush	133 (6.0)	96 (5.6)	37 (7.7)	
Pit	43 (2.0)	29 (1.7)	14 (2.9)	
Open defecation	2032 (92.0)	1604 (92.8)	428 (89.4)	
Mean household size (SD)	7.3 (3.1)	7.4 (3.1)	6.8 (3.0)	< 0.001
Wealth index (%)				0.002
Highest	442 (20.0)	328 (19.0)	114 (23.8)	
Second	442 (20.0)	358 (20.7)	84 (17.5)	
Middle	441 (20.0)	333 (19.3)	108 (22.6)	
Fourth	457 (20.7)	383 (22.2)	74 (15.5)	
Lowest	426 (19.3)	327 (18.9)	99 (20.7)	
Women characteristics				
Mean age (SD) in years	25.5 (4.9)	25.6 (4.9)	25.4 (4.9)	0.429
Attended school (%)	1155 (52.3)	906 (52.4)	249 (52.0)	0.872
Educational level (%)				0.036
Up to primary	404 (35.0)	328 (36.2)	76 (30.5)	
Up to middle	374 (32.4)	299 (33.0)	75 (30.1)	
Up to intermediate	287 (24.9)	217 (24.0)	70 (28.1)	
Others	90 (7.8)	62 (6.8)	28 (11.2)	
Working (%)	457 (20.7)	381 (22.0)	76 (15.9)	0.003
Median number of living children (Q1, Q3)	2 (1, 3)	2 (1, 3)	2 (1, 3)	
Mass media exposure				
Do not read newspaper (%)	1832 (83.0)	1442 (83.4)	390 (81.4)	0.307
Do not listen to radio (%)	1754 (79.4)	1364 (78.9)	390 (81.4)	0.225
Do not watch TV (%)	1384 (62.7)	1091 (63.1)	293 (61.2)	0.439
Access to at least one of the above media (%)	987 (44.7)	772 (44.7)	215 (44.9)	0.927
Marginalization (%)				0.008
Non-marginalized	504 (22.8)	377 (21.8)	127 (26.5)	
Any one form of marginalization	674 (30.5)	534 (30.9)	140 (29.2)	
Any two forms of marginalization	656 (29.7)	504 (29.2)	152 (31.7)	
All forms of marginalization	374 (16.9)	314 (18.2)	60 (12.5)	

### Antenatal care services

According to the guidelines from the government of India, a minimum of four ANCs including early registration and first ANC in the first trimester along with physical and abdominal examinations, haemoglobin (Hb) estimation and urine investigation, two doses of tetanus toxoid (TT) immunization and consumption of iron folic acid (IFA) tablets (6 months during ANC and 6 months during PNC) are required [19]. However, when this survey was done, the recommended minimum number of ANCs was three and the same has been taken for analysis in this paper. Table 2 provides the information on utilization of various maternal health services. It was observed that majority of the pregnancies were registered (89%) and about 83% of women had reported utilization of any ANC services. In order to understand the quality of ANC services, all the components were described separately. Among women who reported utilization of any ANC services, 61% had availed at least three ANCs and only 6% had full ANC (which includes at least three ANC plus two TT injections plus 100+ IFA tablets). Approximately half of the women cited normal pregnancy as the reason for not going for any ANC visit. Other reasons cited were lack of accompanying person or family support, lack of knowledge about antenatal clinics/services and costs involved.

**Table 2:** Utilization of maternal health services during pregnancy, delivery and post-delivery

Variables	Overall *N* = 2208	SHG household (*n* = 1729)	Non-SHG household (*n* = 479)
Antenatal care			
• Pregnancy registered (%)	1972 (89.3)	1554 (89.9)	418 (87.3)
• Utilization of any ANC services (%)	1842 (83.4)	1462 (84.6)	380 (79.3)
At least three ANCs (%)	1126 (61.1)	886 (60.6)	240 (63.2)
Two TT injections (%)	1770 (96.1)	1407 (96.2)	363 (95.5)
• 100 or more IFA tablets (%)	175 (7.9)	134 (7.8)	41 (8.6)
• Full ANC (%)	131 (5.9)	105 (6.1)	26 (5.4)
• Deworming (%)	85 (3.9)	68 (3.9)	17 (3.6)
• Check-ups during pregnancy (%)	(*n* = 1842)	(*n* = 1462)	(*n* = 380)
Urine test	923 (50.1)	723 (49.5)	200 (52.6)
Blood test	774 (42.0)	610(41.7)	164 (43.2)
Blood pressure	704 (38.2)	545 (37.3)	159 (41.8)
Weight	696 (37.8)	538 (36.8)	158 (41.6)
Abdominal examination	1268 (68.8)	1008 (69.0)	260 (68.4)
Ultrasound	577 (31.3)	453 (31.0)	124 (32.6)
• At least three check-ups (%)	526 (28.6)	410 (28.0)	116 (30.5)
• Contact with any health worker (%)	2007 (90.9)	1578 (91.3)	429 (89.6)
Advice on delivery preparedness			
• Delivery preparedness (%)	1240 (56.2)	979 (56.6)	261 (54.5)
• Type of delivery preparedness (%)	(*n* = 1240)	(*n* = 979)	(*n* = 261)
Decision on place of delivery	349 (28.1)	280 (28.6)	69 (26.4)
Arrangement of money for delivery	745 (60.0)	593 (60.6)	152 (58.2)
Arrangement of transport	381 (30.7)	298 (30.4)	83 (31.8)
Identification of institution	107 (8.6)	89 (9.0)	18 (6.9)
Keep clean cloth	939 (42.5)	742 (75.8)	197 (75.5)
Others	513 (75.7)	408 (41.7)	105 (40.2)
Delivery care			
• Type of delivery preparedness (%)	(*n* = 1240)	(*n* = 979)	(*n* = 261)
Decision on place of delivery	289 (23.3)	228 (23.2)	61 (23.4)
Arrangement of money for delivery	817 (65.9)	654 (66.8)	163 (62.4)
Arrangement of transport	380 (30.6)	304 (31.0)	76 (29.1)
Identification of institution	53 (4.3)	49 (5.0)	4 (1.5)
Keep clean cloth	991 (79.9)	792 (80.9)	199 (76.2)
Others	485 (39.1)	400 (40.9)	85 (32.6)
• Institutional delivery (%)	1495 (67.7)	1161 (67.2)	334 (69.7)
• Type of delivery, normal (%)	1349 (90.2)	1061 (91.4)	288 (86.2)
• Duration of stay (h) in facility after delivery			
Median (Q1, Q3)	15 (3, 48)	14 (3, 48)	19 (3, 48)
At least 24 h (%)	674 (45.1)	513 (44.2)	161 (48.2)
At least 48 h (%)	430 (28.8)	325 (28.0)	105 (31.4)
Postnatal care services			
• PNC received (%)	580 (26.3)	462 (26.7)	118 (24.6)
• Median number of PNCs received (Q1, Q3)	1 (1, 2)	1 (1, 2)	2 (1, 2)
1st PNC within a week (%)	381 (65.7)	302 (65.4)	79 (66.9)
Three PNCs within 7 days (%)	71 (12.2)	58 (12.5)	13 (10.9)
Median time of the first PNC in days (Q1, Q3)	5 (1, 12)	4 (1, 12)	5 (2, 12)
• Home visit by health worker within 42 days after delivery (%)	1300 (58.9)	1015 (58.7)	285 (59.5)
Contacted within 7 days (%)	469 (36.1)	351 (34.6)	118 (41.4)
Contacted after 7 days (%)	831 (63.9)	664 (65.4)	167 (58.6)

Coverage of TT immunization was reported by 96% of the women who had taken two TT injections during pregnancy. However, consumption of 100 or more IFA tablets and deworming was reported by 8% and 4% of women respectively. Only 29% of the women had reported at least three important check-ups done during their pregnancy, which includes blood test, blood pressure and abdominal examination.

### Delivery care services

A little more than half of the women were advised by either a field-level health worker or family members on delivery preparedness (Table 2). Field-level functionaries included auxiliary nurse midwife (ANM) or female health workers, accredited social health activists (ASHAs), Anganwari workers (AWW), Swasthya Sakhi, or SHG VO. We found 66% of women or their family had saved or made arrangement of money to meet expenses during delivery or in case of any emergency. About one fourth of them had decided about the place of delivery a priori, and only 30% had arranged for transport in advance to go to the health facility. Identification of institution in case of any complication was reported by only 4% of the women. Among the study participants, 68% of the women delivered in the health facilities out of which only 45% of the women stayed in the facility for at least 24 h. Only about 29% of the women stayed for a minimum duration of 48 h among all the institutional deliveries, which is recommended for the health of the mother and the baby.

### Postnatal care services

Postnatal care allow for identification of emergencies in the immediate postnatal period. Most of the important complications of the postpartum period which can lead to maternal death occur during the first 48 h. This becomes much more important in the case of home deliveries. Even in institutional deliveries, only about one third of the women stayed for 48 h or more (Table 2). Only 26% of all women reported that they had received any PNC within 42 days of delivery, which is approximately the same among SHG and non-SHG households. The median number of PNCs within 42 days of delivery was one, less than what is recommended. Among those women who received PNC, 66% had their PNC within a week after delivery; however, only 12% reported having three PNCs within a week. Home visit by any of the health worker within 42 days of delivery was reported by 59% of the women.

### Danger signs and complications during pregnancy, delivery and postnatal period

Recognition of danger signs by the women during pregnancy, delivery and postnatal period is crucial for timely action and management. Knowledge of the danger signs during pregnancy, delivery and post-delivery among the women was very low (Table 3). The median number of danger signs that the women remembered was two. Despite their poor knowledge, majority (more than 80%) of the women who reported having complications had sought treatment for the same.

**Table 3:** Knowledge of danger signs, experience of complications and sought treatment during pregnancy, delivery and post-delivery

Key danger signs	Knowledge of danger sings	Complications during pregnancy	Sought treatment^a^
During pregnancy	(n = 2208)	(n = 1190)	
Median number of complications they are aware of (Q1, Q3)(min, max)	2(2, 3)(1, 9)		
Excessive vaginal bleeding	324 (14.7)	51 (4.3)	48 (94.1)
Severe headache	191 (8.7)	39 (3.3)	31 (79.5)
Vaginal discharge (foul smelling)/water	276 (12.5)	87 (7.3)	84 (96.6)
Blurred vision/visual disturbance	148 (6.7)	45 (3.7)	23 (52.3)
Convulsions	21 (0.9)	4 (0.3)	4 (100.0)
High blood pressure	44 (2.0)	10 (0.8)	10 (100.0)
Swelling of feet/hand	564 (25.5)	188 (15.8)	121 (64.4)
Severe lower abdominal pain	1187 (53.4)	491 (41.2)	396 (80.7)
During delivery		(n = 728)	
Median number of complications they are aware of (Q1, Q3) (min, max)	2(1, 3)(1, 13)		
Excessive vaginal bleeding	761 (34.5)	116 (15.9)	97 (83.6)
Feel something discharge	256 (11.6)	61 (8.4)	55 (90.2)
Severe abdominal pain	920 (41.7)	396 (54.4)	326 (82.3)
Prolonged labor over 12 h	219 (9.9)	67 (9.2)	55 (82.1)
Baby in abnormal position	651 (29.5)	62 (8.5)	59 (95.2)
Cord around the neck	249 (11.3)	24 (3.2)	23 (95.8)
Convulsions	55 (2.5)	8 (1.1)	7 (87.5)
High fever	247 (11.2)	30 (4.1)	28 (93.3)
Post-natal period		(n = 658)	
Median number of complications they are aware of (Q1, Q3) (min, max)	2 (1, 3) (0, 7)		
Excessive vaginal bleeding	707 (32.0)	97 (14.7)	76 (78.4)
Severe headache	217 (9.8)	52 (7.9)	46 (88.5)
Foul smelling vaginal discharge	135 (6.1)	24 (3.6)	19 (79.2)
Convulsions or fits	36 (1.6)	6 (0.9)	5 (83.3)
High fever	824 (37.3)	238 (36.2)	234 (98.3)
Severe abdominal pain	674 (30.5)	188 (28.6)	158 (84.0)
Blurred vision	130 (5.9)	39 (5.9)	30 (76.9)
Breast related problems	43 (1.9)	16 (2.4)	15 (93.8)

^a^Denominator (*n*) for each cell of this column is those who reported complications in the previous column

### Determinants of utilization of maternal health care services

In order to obtain determinants of at least three ANC visits, place of delivery and PNC, the covariates considered were age of the women, type of family, working status of the women, mass-media exposure, number of contacts with the health worker during ANC period, SHG membership and marginalization. Univariate analysis was done to find out the association with the outcome variables. Irrespective of whether they were associated or not, those were included in the multivariable model. Multilevel random intercept logistic regression was carried out for each of the three outcomes separately to adjust the effect of clustering at GP and block levels. Besides these variables, few additional covariates were also included considering their biological plausibility. For example, at least three ANC visits were controlled for place of delivery outcome. Additional covariates that were adjusted for PNC outcome were at least three ANC visits and place of delivery. Results of both adjusted and unadjusted analyses are presented in Table 4.

**Table 4:** Determinants of utilization of maternal health services during pregnancy, delivery and post-delivery

Variables	At least three ANCs (ORs, 95% CI)		Place of delivery (ORs, 95% CI)		PNC within 42 days (ORs, 95% CI)	
	Unadjusted	Adjusted	Unadjusted	Adjusted	Unadjusted	Adjusted
Age of the women	0.98 (0.96, 1.00)	0.98 (0.96, 1.01)	0.94 (0.92, 0.96)	0.97 (0.95, 0.99)	0.98 (0.96, 0.99)	1.01 (0.98, 1.03)
*p* value	0.026	0.176	< 0.001	0.026	0.026	0.646
Type of family						
Non-nuclear	1	1	1	1	1	1
Nuclear	0.87 (0.71, 1.06)	1.01 (0.81, 1.26)	0.65 (0.53, 0.79)	0.82 (0.65, 1.05)	0.71 (0.58, 0.88)	0.91 (0.71, 1.16)
*p* value	0.161	0.939	< 0.001	0.115	0.002	0.437
Working						
No	1	1	1	1	1	1
Yes	0.85 (0.66, 1.10)	0.92 (0.70, 1.20)	0.66 (0.52, 0.84)	0.87 (0.66, 1.16)	0.98 (0.75, 1.26)	1.33 (0.98, 1.80)
*p* value	0.216	0.529	0.001	0.352	0.848	0.064
Mass-media exposure						
No	1	1	1	1	1	1
Yes	1.70 (1.39, 2.08)	1.52 (1.22, 1.90)	1.43 (1.17, 1.75)	0.91 (0.71, 1.16)	1.57 (1.27, 1.94)	1.23 (0.96, 1.58)
*p* value	< 0.001	< 0.001	0.001	0.449	< 0.001	0.097
Number of contact with health worker during ANC period	1.10 (1.07, 1.13)	1.11 (1.08, 1.14)	1.03 (1.00, 1.06)	1.01 (0.98, 1.04)	1.05 (1.02, 1.08)	1.04 (1.01, 1.07)
*p* value	< 0.001	< 0.001	0.044	0.573	0.001	0.011
SHG membership						
No	1	1	1	1	1	1
Yes	0.91 (0.70, 1.19)	0.87 (0.66, 1.15)	1.03 (0.80, 1.34)	1.05 (0.78, 1.42)	1.13 (0.86, 1.48)	1.08 (0.80, 1.46)
*p* value	0.493	0.332	0.807	0.747	0.392	0.602
Marginalization						
Non-marginalized	1	1	1	1	1	1
Any one form of marginalization	0.68 (0.52, 0.90)	0.75 (0.56, 0.99)	0.50 (0.36, 0.68)	0.51 (0.36, 0.72)	0.73 (0.55, 0.97)	0.73 (0.54, 0.97)
Any two form of marginalization	0.55 (0.41, 0.73)	0.62 (0.45, 0.85)	0.26 (0.19, 0.36)	0.33 (0.23, 0.48)	0.53 (0.38, 0.72)	0.52 (0.37, 0.73)
Most marginalized	0.53 (0.37, 0.74)	0.69 (0.47, 1.02)	0.23 (0.16, 0.33)	0.29 (0.19, 0.46)	0.59 (0.40, 0.87)	0.59 (0.38, 0.92)
*p* value	< 0.001	0.032	< 0.001	< 0.001	< 0.001	0.003
At least three ANCs^a^						
No			1	1	1	1
Yes			1.67 (1.34, 2.10)	1.55 (1.23, 1.96)	1.80 (1.41, 2.29)	1.52 (1.19, 1.95)
*p* value			< 0.001	< 0.001	< 0.001	0.0.001
Place of delivery^b^						
Home					1	1
Institution					2.67 (2.05, 3.47)	2.43 (1.81, 3.27)
*p* value					< 0.001	< 0.001

^a^Exposure for place of delivery and PNC outcome

^b^Exposure for PNC outcome

In the adjusted analysis, women with more number of contacts with the health worker during the ANC period (OR = 1.11, 95% CI = 1.08, 1.14, *p* value < 0.001) and exposed to mass media (OR = 1.52, 95% CI = 1.22, 1.90, *p* value < 0.001) were more likely to have at least three ANC visits during pregnancy. Most marginalized women were less likely to avail ANC visits (OR = 0.69, 95% CI = 0.47, 1.02, *p* value = 0.032) as compared to non-marginalized women.

Women with all forms of marginalization were less likely to deliver in the health facility as compared to non-marginalized women after controlling for other covariates (OR = 0.29, 95% CI = 0.19, 0.46, *p* value < 0.001). It was observed that with increasing age, odds of delivering in an institution reduced (OR = 0.97, 955 CI = 0.95, 0.99, *p* value = 0.026). Further, we found that women who had at least three ANC visits (OR = 1.67, 95% CI = 1.34, 2.10, *p* value < 0.001) were more likely to go for institutional delivery as compared to their counterparts.

The analysis revealed that women with an increasing number of contacts with health workers (OR = 1.04, 95% CI = 1.01, 1.07, *p* value = 0.011), at least three ANC visits (OR = 1.53, 95% CI = 1.20, 1.97, *p* value = 0.001) and institutional delivery (OR = 2.48, 95% CI = 1.84, 3.34, *p* value < 0.001) were more likely to avail any PNC within 42 days. Most marginalized women were less likely to attend any PNC within 42 days (OR = 0.59, 95% CI = 0.38, 0.92, *p* value = 0.003) as compared to non-marginalized women.

No association was observed between the utilization of ANC, institutional delivery and PNC with SHG membership after controlling for the covariates.

## Discussion

Utilization of maternal health services which includes antenatal services, institutional delivery and postnatal services was low in the study population. Number of contacts with the health worker in the ANC period and mass-media exposure was strongly associated with utilization of ANC and PNC services. Marginalization of women emerged as a strong determinant for utilization of maternal health services. SHG membership did not seem to be associated with any of the outcomes.

It was observed in our study that 83% of the women had utilized any ANC services and among them 61% reported at least three ANCs during pregnancy. These proportions are high when compared to the overall figure of UP. According to National Family Health Survey-4 (NFHS-4), about 22% of mothers had at least four ANC visits in the rural area of UP [20]. One reason could be because of the change in guidelines from a minimum of three to four antenatal visits. Our finding of any ANC is similar to what has been reported in other studies from India and UP [21, 22]. However, varied proportions (86–42%) for at least three ANCs were reported by different national- and local-level studies in India [21–23]. In the present study, only 6% of the women had full ANC similar to what is reported in the NFHS-4 (4%) [20]. A study done in different states of India reported the coverage of full ANC in UP as 35% [21] which is quite high as compared to what we found in this study.

The consumption of IFA tablets was very low (7.9%) in our study which is very much comparable to the findings of NFHS-4 in the rural area of UP (10.9%) [20]. This proportion is lower than the national figure in rural areas (25.9%) [24]. It emerged from the analysis that TT injection during pregnancy was almost universal in the study area in contrast with the ANC utilization (83%). This suggests that women might have gone to other centres for taking TT injections or went to receive only TT injection and did not receive antenatal check-up.

Although utilization of ANC services was more than 60%, yet the quality of services remains suboptimal. This is reflected from the proportion of women who reported consumption of IFA tablets and examined for blood pressure, weight gain and blood and urine test. Similar findings were also reported by the Annual Health Survey (AHS) where important check-ups during pregnancy like blood pressure, Hb measurement and ultrasound (35%, 27% and 31%, respectively) were very low [25]. Similar findings of high coverage but low quality of ANC services are being reported from different countries [26, 27]. One of the reasons for low proportion of women getting quality ANC could be the absence of adequate infrastructure and instruments at the facility [27].

In our study, we observed that delivery preparedness in terms of arrangement of money and clean clothes was fairly good but less proportion of women were well prepared on other important parameters like identification of institution and transport arrangement in case of emergency. Similar findings were reported in other studies from India and abroad [28–30]. Preparedness regarding identification of health facility or skilled attendant during delivery was reported to be high in some studies [29, 30] in contrast to what was observed in this study. We observed that only 4% of the women in the study had identified the health facility for any complication that might arise; it could be because of lack of sensitization of pregnant mothers during pregnancy about delivery preparedness.

Institutional delivery in the survey area was 68% which is similar to reports from rural areas of UP (67% as per NFHS-4) [20] but less than the national rural figure of India (75%) [24]. Other studies from North India have reported proportion of institutional deliveries ranging from 54% in rural Madhya Pradesh [31] to 79% in rural area of Jammu [32]. Stay in the facility for an adequate time after the delivery is crucial both for the mother and the baby. This study revealed that about half of the women (55%) had not stayed even for 24 h and nearly one fourth stayed for at least 48 h after the delivery. However, it was reported in AHS (2012–2013) that around 77% of mothers received PNC within 48 h of delivery which is quite high when compared to the findings of this study [25].

PNC is crucial for the health of the newborn as studies have found that neonatal deaths were significantly lower when facility delivery is combined with postnatal check-ups [33]. We observed in our study that the utilization of any PNC services within 42 days of delivery was low (26%). Utilization of PNC is a point of concern in rural UP, since the latest NFHS-4 also reported that approximately 52% of women received PNC from a doctor/nurse/ANM/midwife/LHV (lady health visitor or health supervisor) or other health personnel within 2 days of delivery [20]. Similar findings are reported from other developing countries [34, 35]. Studies have shown that PNC at home by the health worker can also have an impact on the survival of the baby. A cluster randomized trial done in Haryana, having the training of community health worker for postnatal home visits as an intervention, observed that infant mortality and neonatal mortality beyond 24 h were lower in the intervention area as compared to those in the control area [36]. Although 59% of the health worker had visited the household during the postnatal period, only 26% had reported having received PNC services. This discrepancy could be probably because the health worker who visited the newborn might not have conducted a postnatal check-up of the mother. Hence, apart from the PNC at the hospital, emphasis should be given to the health care worker for doing PNC during home visits.

Mass-media exposure, number of contacts with health workers and marginalization emerged as significant determinants for at least three ANCs in our analysis. Other studies have also shown a positive influence of exposure to mass media, like radio and television, on the utilization of antenatal care services [37, 38]. Socio-economic status, caste and education of women were reported as the important determinants of availing ANC services [9, 32, 39, 40] which is consistent to the relationship we observed here with marginalization (which includes education, caste and household wealth).

Age, marginalization and at least three ANC visits were found to be the significant predictors for institutional delivery in this study. Previous studies have also shown a positive association between socio-economic status and skilled attendance at delivery [5, 41]. Cost could be a probable reason for low institutional deliveries among the most marginalized group. It was reported in other studies that likelihood of delivering in an institution increases when women had availed adequate ANC visits similar to the findings of this study [9, 42].

Positive influence of contacts with health workers, marginalization, at least three ANC visits and institutional delivery was observed for any PNC within 42 days in the multivariable analysis. Similar findings also emerged from many other studies in India and other developing regions [35, 40, 43, 44]. The direction of association was also similar to what has been observed in various studies except the study from Nigeria, where it was observed that the likelihood of utilization of PNC services was more if women delivered at home [35].

In our analysis, we could not find any association of at least three ANC visits, institutional delivery and PNC with SHG membership. This could be because health was never discussed during their meetings. Women might be using SHGs for financial purposes primarily. Hence, this platform of existing SHGs can be utilized to spread health awareness especially related to women and child. A recent study from two states of India showed improved outcomes in terms of institutional deliveries and feeding colostrum to their newborns, if health programmes are implemented with microfinance-based groups like SHGs [45].

The major strengths of this study lie in its design, as this was a community-based cross-sectional study with a fairly large sample size. The survey was done covering a larger area of 15 blocks from five districts of UP, which further add to the representativeness of the selected population. Most studies have reported the indicators like education, caste and socio-economic status separately as determinants. However, in the present study, we have used these variables and created a composite indicator, marginalization, which gives a better picture. The survey was done by a group of trained people under strict supervision. Besides these strengths, there are certain limitations as well in the study. The population covered in the survey was mainly the marginalized section from rural areas with low education and from other backward caste (OBC)/SC/ST; hence, the generalization of the findings to the larger population would be questionable. Although the length of recall period in the study was up to 15 months only to avoid any kind of recall bias, still there may be some recall bias. Absence of documents for verifying the given maternal health information further compounds the problem.

## Conclusion

It was observed that utilization of maternal health services during pregnancy, delivery and post-delivery was low in the study population, especially the postnatal care. Utilization of antenatal services was an important determinant both for institutional delivery and postnatal care. Mass-media exposure was positively associated with the utilization of maternal health care services during pregnancy and post-delivery, hence efforts should continue to provide health messages using different media including television and radio. For both antenatal care and postnatal care, role of health worker was crucial as it was observed that women were more likely to avail ANC and PNC with increasing number of contacts with the health workers. Most marginalized women were less likely to avail these services during pregnancy, delivery and post-delivery; hence, interventions should focus on these populations. Even though SHG membership was not associated with the utilization of maternal health care services in the study, yet these SHGs can be used for delivering health care messages within and beyond the group, which is the aim of the main intervention study.

## Data Availability

The datasets analysed during the study are available from the corresponding author on reasonable request and after approval of the concerned authorities in the organization.

## Abbreviations

AHS: Annual Health Survey
ANC: Antenatal care
CI: Confidence interval
GPs: Gram panchayats
Hb: Haemoglobin
IFA: Iron folic acid
MMR: Maternal mortality ratio
NFHS: National Family Health Survey
OBC: Other backward caste
OR: Odds ratio
PNC: Postnatal care
RGMVP: Rajiv Gandhi Mahila Vikas Pariyojana
SC/ST: Scheduled caste/scheduled tribe
SES: Socio-economic status
SHG: Self-help group
TT: Tetanus toxoid
UP: Uttar Pradesh
